# Effects of a group-based lifestyle medicine for depression: A pilot randomized controlled trial

**DOI:** 10.1371/journal.pone.0258059

**Published:** 2021-10-08

**Authors:** Agnes Ka-Yan Ip, Fiona Yan-Yee Ho, Wing-Fai Yeung, Ka-Fai Chung, Chee H. Ng, Georgina Oliver, Jerome Sarris

**Affiliations:** 1 Department of Psychology, The Chinese University of Hong Kong, Shatin, Hong Kong; 2 School of Nursing, The Hong Kong Polytechnic University, Hunghom, Hong Kong; 3 Department of Psychiatry, The University of Hong Kong, Pokfulam, Hong Kong; 4 Department of Psychiatry, Professorial Unit, The Melbourne Clinic, The University of Melbourne, Melbourne, Victoria, Australia; 5 Western Sydney University, NICM Heath Research Institute, Westmead, NSW, Australia; Prince Sattam Bin Abdulaziz University, College of Applied Medical Sciences, SAUDI ARABIA

## Abstract

Given the growing evidence that a range of lifestyle factors are involved in the etiology of depression, a ‘lifestyle medicine’ approach can be potentially safe and cost-effective to prevent or treat depression. To examine the effects and acceptability of a group-based, integrative lifestyle medicine intervention as a standalone treatment for managing depressive symptoms, a pilot randomized controlled trial (RCT) was conducted in a Chinese adult population in 2018. Participants (*n* = 31) with PHQ-9 score above the cut-off of ≥ 10, which was indicative of moderate to severe depression, were recruited from the general community in Hong Kong and randomly assigned to lifestyle medicine group (LM group) or care-as-usual group (CAU group) in a ratio of 1:1. Participants in the LM group received 2-hour group sessions once per week for six consecutive weeks, which covered diet, exercise, mindfulness, psychoeducation, and sleep management. Linear mixed-effects model analyses showed that the LM group had a significant reduction in PHQ-9 scores compared to the CAU group at immediate posttreatment and 12-week posttreatment follow-up (*d* = 0.69 and 0.73, respectively). Moreover, there were significantly greater improvements in anxiety, stress, and insomnia symptoms (measured by DASS-21 and ISI) at all time points in the LM group (*d* = 0.42–1.16). The results suggests that our 6-week group-based, integrative lifestyle intervention program is effective in lowering depressive, anxiety, stress, and insomnia symptoms in the Chinese population. Further studies in clinical populations with a larger sample size and longer follow-up are warranted.

## Introduction

Depression is a common mental disorder which is characterized by a prolonged depressed mood, loss of interest, disturbed sleep or appetite, feelings of worthlessness or guilt, poor concentration, and recurring thoughts of suicide [1]. Depression significantly reduces an individual’s quality of life and is associated with higher mortality [2]. It was estimated that over 300 million people suffered from depression, equivalent to 4.4% of the world’s population [3]. A recent 10-year prospective cohort study by Ni et al. suggested that the prevalence of probable depression had risen from 6.5% in 2017 to 11.2% in 2019 in the Hong Kong adult population [4]. According to the World Health Organization [5], depression has a major impact on health and society and is projected to be one of the three leading causes of global disease burden by 2030 [6].

Lifestyle factors are increasingly recognized as important to mental health, in which healthy lifestyles have a positive impact on improving depression and anxiety as shown in prospective studies [7, 8]. Some researchers have begun to explore the effectiveness of lifestyle medicine (LM) for promoting mental health. LM is a relatively new medical specialty, which is generally defined as the prevention and treatment of disease by changing personal habits and behavioral choices of individuals in order to address the underlying causes [9]. In the past, LM mainly focused on the prevention, management, and treatment of physical diseases, such as heart disease and diabetes [10, 11]. Within the last decade, growing evidence has found that depression has a significant lifestyle-driven component [12–15]. For example, lifestyle factors, such as diet, exercise, and sleep, are found to be significant mediating factors for the development, progression, and treatment of depression [13]. A review by Berk and colleagues (2013) also reported that lifestyle factors, such as poor diet, sedentary lifestyle, smoking and substance abuse, contribute to depression risk [16]. According to the review by Sarris et al., lifestyle elements that have sound evidentiary support for managing depression include good dietary and sleep quality, adequate physical activity and mindfulness-based practice, and avoidance of harmful substances (e.g., smoking, alcohol, or illicit substances) [14].

Specifically, recent systematic reviews and meta-analyses demonstrated that healthy diet patterns, usually characterized by high intakes of fruit, vegetables, fish, and whole grains, were significantly associated with a reduced depression risk [17–20]. A meta-analysis conducted by Firth et al. demonstrated that dietary interventions significantly reduced depressive symptoms [21]. The effect of physical activity as a treatment for depression was reported in several reviews [22–24]. In a recent meta-analysis of 27 systematic reviews (including 16 meta-analyses representing 152 RCTs) which investigated exercise interventions with aerobic exercise, high-intensity exercise, and resistance training, reported that exercise reduced depression in patients with major depressive disorder (MDD) across different age groups [25]. Moreover, studies also demonstrated that the effect of exercise as a treatment for depression was comparable to first-line treatments, such as medications and cognitive-behavioral therapy (CBT) [26–28]. Treatment for insomnia is usually recommended as part of the treatment for depression, given the high co-occurrence of insomnia and depression [29]. A review study of several meta-analyses demonstrated that CBT for both insomnia and depressive symptoms was safe and effective, and its efficacy exerted stable long-term effects as evidenced by longitudinal follow-ups [30]. Mindfulness is another lifestyle element that has growing evidence for its efficacy in treating depression. A comprehensive meta-analysis of RCTs of Mindfulness-Based Interventions (MBIs) in patients who experienced a current episode of MDD reported that participants receiving MBIs showed a significant reduction in depressive symptoms at post-intervention assessment, compared to the control group [31]. Another meta-analysis of 142 RCTs demonstrated strong evidence that the effects of MBI were equivalent to those of existing evidence-based treatments for depression [32].

Supported by the growing evidence which acknowledges the significance of lifestyle factors associated with depression, it is proposed that LM could offer a potentially safe and cost-effective treatment option for the prevention, management, and treatment of depression [16, 33]. Nonetheless, existing studies on lifestyle modifications for depression are largely limited to individual lifestyle components or either cross-sectional or longitudinal data. Thus, interventional clinical trials using a multi-component integrative LM approach for depression is very limited. Previous RCTs have only examined the effects of LM interventions delivered merely through written recommendations [34, 35]. Other RCTs were conducted among depressed older-adults through individual calls and visits [36, 37]. Recently, some researchers started to develop integrated lifestyle programs, such as Healthy Body Healthy Mind (HBHM) [38]. However, there is no RCT investigating a group-based, integrative LM intervention in the adult population for depression.

To the best of our knowledge, our study was the first pilot RCT of an integrative, group-based lifestyle intervention for depressed adults not on current treatment. The aims of this study were to (1) examine the effects and acceptability of an integrative LM intervention for the management of depressive symptoms in a Chinese adult population and (2) inform the study design and sample size calculation for future fully powered trials.

## Materials and methods

### Study design

This prospective study was a pilot RCT on the effects of lifestyle medicine as a standalone treatment for managing depressive symptoms. Thirty-one eligible participants were randomly assigned to either the lifestyle medicine group (LM group) or the care-as-usual group (CAU group) in a ratio of 1:1. The study was reviewed and approved by the Survey and Behavioural Research Ethics Committee (SBREC) of the Chinese University of Hong Kong (Reference No. SBRE-18-055). The trial was registered in ClinicalTrials.gov (identifier: NCT03720145), and the RCT followed Consolidated Standards for Reporting Trials (CONSORT) guidelines for non-pharmacological interventions.

### Participants

Participants were recruited from the community through social media sites and university mass mails from October 2018 to November 2018. Eligible participants were screened on the basis of the following inclusion criteria: (1) aged 18 or above; (2) Hong Kong residents with proficiency in Cantonese; (3) Patient Health Questionnaire (PHQ-9) score above the cut-off of ≥ 10 [39]; and (4) a willingness to provide informed consent and comply with the trial protocol. Participants were excluded if they (1) were pregnant; (2) had suicidal ideations based on the Beck Depression Inventory (BDI-II) Item 9 score ≥ 2 (referral information to professional services was provided to those who endorsed items on suicidal ideation); (3) were using medication or psychotherapy for depression; or (4) were diagnosed with having any major psychiatric, medical or neurocognitive disorder(s) that made participation unsuitable, or interfered with the adherence to the lifestyle modification where exercise or a change in diet were not recommended by physicians.

### Study procedure

Potential participants were enrolled by providing online informed consent and completing an online screening survey using the Qualtrics platform. Eligible participants were then contacted by a research assistant to explain the screening results and study procedures and confirm their availability to participate in the group treatment. Those who were available for the study were randomly assigned to either the LM group or the CAU group. Block randomization with a block size of 4 was performed by an independent assessor using a computer-generated list of numbers in Excel. Allocation concealment was achieved by storing the randomization sequence in a secure electronic file which was inaccessible to the study investigators and therapists. Due to the study nature, blinding of participants and therapists was not possible. Participants in the LM group received the 2-hour group lifestyle intervention at the Chinese University of Hong Kong once per week for 6 consecutive weeks from November to December of 2018. They did not receive other care during the study period; however, referral was provided to those who need more intensive interventions based on the team’s clinical judgment. The CAU group did not receive the lifestyle intervention but continued receiving care as usual, defined broadly as anything patients would normally receive. There were no restrictions regarding the care they received during the study. A list of mental health services available in the community (e.g., regional psychiatric centres and non-governmental organizations), public hospitals, and emergency contact were provided to the participants. Both the LM group and the CAU group completed the same set of online questionnaires at 3 time points: baseline (Week 0), immediate posttreatment (Week 6), and 12-week posttreatment follow-up (Week 18). All data were collected using an in-house smartphone application called Longitudinax. For the participants who have completed all outcome assessments, a cash incentive of HKD 100 was offered for each participant after completing all study procedures.

### Intervention

The intervention protocol was modified and enhanced from the Healthy Body Healthy Mind (HBHM) integrative lifestyle program, which was designed to integrate five evidence-based lifestyle components, including diet, exercise, mindfulness, psychoeducation, and sleep management, alongside motivational and goal setting skills and lifestyle psychoeducation [38]. The program consisted of 2-hour group sessions conducted in person once per week for six consecutive weeks. Moreover, the intervention protocol used in this pilot study was adapted to the local culture and lifestyle, particularly on diet. For example, Chinese dietary recommendations were primarily based on Guangdong dishes, soups, and herbal tea. Handouts which consisted of treatment content of each session, exercises (e.g., QR codes that linked to relaxation and mindfulness), and record forms were distributed to the LM group and the CAU group during each session and at the end of the study, respectively. The content outline is summarized in S1 Table. The group treatment was primarily delivered by a clinical psychology trainee under the on-site supervision of a clinical psychologist. Other instructors included a dietitian, a physical instructor, and a Chinese medicine practitioner. Prior to the beginning of and during the 6-week treatment period, a research assistant encouraged treatment compliance and daily practices of lifestyle modifications by sending text reminders to participants between sessions.

### Measures

The self-administered questionnaires assessed depressive symptoms, insomnia severity, fatigue, quality of life, daytime functioning, and treatment evaluation. The following instruments were included after a review of appropriateness based on their content validity, reliability, and the availability of the Chinese-language version.

#### Sociodemographic data

The background questionnaire collected age, gender, marital status, education level, current employment status, mental health and physical health history, and lifestyle-related characteristics (e.g., smoking, drinking, physical exercise, and meditation).

#### The Patient Health Questionnaire

The primary outcome was measured by the Patient Health Questionnaire (PHQ-9) [40], which is a 9-item questionnaire used for screening, diagnosing, monitoring, and measuring the severity of depression. It scores each of the nine diagnostic criteria of the Diagnostic and Statistical Manual of Mental Disorders, 4^th^ Edition (DSM-IV) for depression as “0” (not at all) to “3” (nearly every day) to assess depression and suicidal ideation in the past 2 weeks [39]. A total score of 10 or above, which was set as one of the inclusion criteria in the current study, indicates the presence of probable depression of moderate to severe levels. The Chinese version of PHQ-9 has been validated in Hong Kong and was found to have a sensitivity of 80% and a specificity of 92% for diagnosing major depression [41]. For this study, a PHQ-9 score of 10 or above was used to identify depressive symptoms.

#### The Depression Anxiety Stress Scales

The Depression Anxiety Stress Scale-21 (DASS-21) is a 21-item scale [42, 43], comprises three sub-scales which measures depression, anxiety, and stress, over the past week on a 4-point Likert scale from 0 (never) to 3 (almost always). Scores are summed over 7-item Depression, Anxiety and Stress sub-scales. The total scores indicate the level of severity (normal, mild, moderate, severe, and extremely severe). The Chinese version of DASS-21 was validated in the Hong Kong Chinese population among clinical and non-clinical samples [43, 44].

#### Insomnia Severity Index

The Insomnia Severity Index (ISI) is a 7-item scale to measure the severity of insomnia symptoms and the associated daytime impairment on a 5-point Likert scale [45]. Ratings are obtained on the perceived severity of sleep-onset, sleep-maintenance, early morning awakening problems, satisfaction with current sleep pattern, interference with daily functioning, noticeability of impairment attributed to the sleep problem, and level of distress caused by the sleep problem. The Chinese version of ISI was validated with good internal consistency (Cronbach’s alpha = 0.83) and 2-week test–retest reliability (Pearson’s *r* = 0.79) [46].

#### Short-Form 6-Dimension

Short-Form 6-Dimension (SF-6D) is used to measure the quality of life of an individual using the six-level preference-based assessment derived from the health survey SF-36 [47]. The Chinese version of SF-6D was adopted for cost-utility analyses, which included physical and social functioning, role participation, mental health, bodily pain, and vitality. The theoretical range of SF-6D utility score ranged from 0.315 for the worse possible health state to 1 for full health according to the Chinese Hong Kong population-specific scoring algorithm [48]. The Chinese SF-6D showed good test-retest reliability and an intra-class correlation of 0.79 [49].

#### Multidimensional Fatigue Inventory

Multidimensional Fatigue Inventory (MFI) is a 20-item self-report instrument designed to measure fatigue [50]. Ratings on a 5-point Likert scale are obtained on the dimensions of general fatigue, physical fatigue, mental fatigue, reduced motivation, and reduced activity. Ten positively phrased items (item 2, 5, 9, 10, 13, 14, 16, 17, 18, 19) were reverse-scored before adding up scores. The scores ranged from 20 to 100, with higher scores indicating higher levels of fatigue. The Chinese version showed good internal consistency (Cronbach’s α = 0.89) and a test–retest reliability of 0.73 [51].

#### Sheehan Disability Scale

Sheehan Disability Scale (SDS) is a brief self-report tool that assesses functional impairment in work/school, social life, and family life. On each of the domains, functioning is rated from 0 to 10 (0: no impairment; 1–3: mild impairment; 4–6: moderate impairment; 7–9: marked impairment; 10: extreme impairment) [52]. The level of global functioning is determined using the sum of the three domains. Higher scores mean greater disability/lower functioning. The Chinese SDS showed good internal consistency (Cronbach’s α = .89) and moderate test–retest reliability (intraclass correlation coefficient = 0.55) [53].

#### Credibility/Expectancy Questionnaire

The Credibility/Expectancy Questionnaire (CEQ) is a 6-item measure that is commonly used in RCTs of behavioral interventions [54]. It comprises two subscales: Credibility and Expectancy. The credibility subscale measures beliefs regarding the strength of the treatment. In the current study, the overall credibility rating was calculated by taking the mean of the first three items of the CEQ. The expectancy subscale measures the extent to which participants feel their symptoms will improve during the intervention. In the current study, treatment credibility and expectancy were collected from both groups before the intervention, and acceptability and satisfaction were collected from the LM group after the intervention using the CEQ. Expectancy ratings were measured by examining the final three items of the CEQ individually. The CEQ has good evidence of strong internal consistency and good test-retest reliability across clinical populations. The Cronbach’s alphas were .75 for credibility and .95 for expectancy. The CEQ demonstrated good internal consistency (Cronbach’s α = 0.84) and test-retest reliability (0.82 for expectancy and 0.75 for credibility) [54].

### Statistical analysis

The sample size calculation of the current study followed the recommendation that a minimum 12 participants per group be considered for pilot clinical trials [55]. Such recommendation was justified based on rationales about feasibility and precision around the estimates. Statistical analyses were conducted using SPSS Windows 22.0 (IBM SPSS Inc., Chicago, IL). An intention-to-treat approach was used for all analyses. Statistical significance was set at 0.05 (2-sided). Between-group effect sizes were calculated using Cohen’s *d*, in which effect sizes of 0.2, 0.5, and 0.8 were considered small, medium, and large effects, respectively. The scoring of the questionnaires was performed according to the corresponding scoring manuals. Between-group differences in baseline characteristics were not computed according to the recommendation of the CONSORT statement. Clinically significant improvement was defined as 50% reduction of PHQ-9 score or 5-point drop from baseline PHQ-9 score at the posttreatment assessments. The between-group difference in the proportion of participants who attained clinically significant improvement was performed by the Fisher’s Exact Test. Linear mixed-effects models (LMM) were used to compare the treatment effect between the LM group and CAU group from baseline (Week 0) to immediate posttreatment (Week 6) and 12-week follow-up assessments (Week 18). Fundamental assumptions of LMM (e.g., normality, validity of the model, independence of data points) were tested to ensure the accuracy of test results. In the sensitivity analysis, only participants who had completed at least 80% of the sessions (i.e., at least 5 out of 6 sessions) were included for testing the effects of the lifestyle intervention. Treatment credibility and expectancy in the LM group from the baseline to immediate posttreatment assessment was examined by paired *t*-test.

## Results

### Baseline characteristics of participants

**Fig 1:**
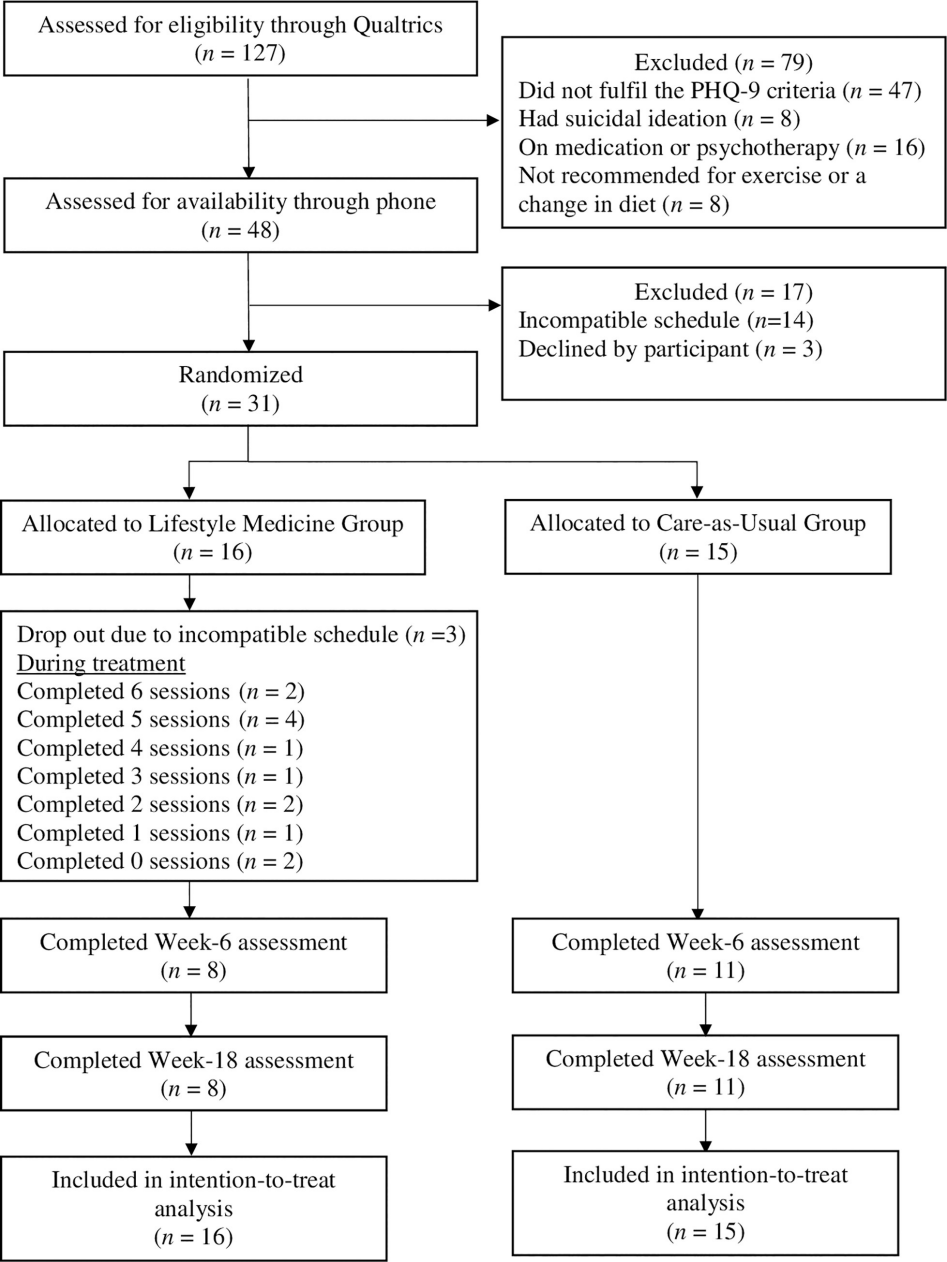
CONSORT study flowchart.

Baseline characteristics and the CONSORT study flowchart are summarized in S2 Table and Fig 1 respectively. A total of 127 individuals completed the informed consent and online screening, and 79 were found to be ineligible primarily due to PHQ-9 < 10 (*n* = 47) and currently on psychotherapy and/or medication (*n* = 16). Details of the exclusion are listed in Fig 1. They were then contacted by phone to assess their availability, and 31 of them agreed to participate in the study. They were randomized to the LM group (*n* = 16) vs. the CAU group (*n* = 15). Of these 31 participants, 5 (16.1%) were males and 26 (83.9%) were females. The mean age of these participants was 35.1 with a *SD* of 15.4 years. Eight of them (25.8%) had completed secondary education and 21 of them (67.7%) university or above education. Their average PHQ-9 score was 12.6 ± 3.1 (placing them at a moderate level of depression).

### Treatment effects

S3 Table presents the statistical summary of outcome measures from the baseline to immediate posttreatment (Week 6) and 12-week follow-up (Week 18). The linear mixed-effects model analysis demonstrated that the PHQ-9 score was reduced significantly more in the LM group than the CAU group from the baseline to immediate posttreatment (*p* = .02, *d* = 0.69), and also at 12-week follow-up (*p* = .01, *d* = 0.73) assessment, both with medium effect sizes. Moreover, the LM group was also superior to the CAU group in improving DASS–Depression (immediate posttreatment: *p* = .008, *d* = 0.98; 12-week follow-up: *p* = .008 .01, *d* = 0.45), DASS–Anxiety (immediate posttreatment: *p* = .03, *d* = 1.16; 12-week follow-up: *p* = .03, *d* = 0.77), DASS–Stress (immediate posttreatment: *p* = .04, *d* = 0.42; 12-week follow-up: *p* = .04, *d* = 0.65), and ISI (immediate posttreatment: *p* = .048, *d* = 0.78; 12-week follow-up: *p* = .046, *d* = 0.93) from the baseline to immediate posttreatment and 12-week follow-up assessments, with small to large effect sizes. However, no significant group × time differences were found in MFI-20, SF-6D, and SDS across the time points (*p* = .09 –.35).

### Clinical response

At immediate posttreatment assessment (Week 6), an insignificant difference was found between the LM group and the CAU group in the proportion of participants who attained 50% reduction from baseline PHQ-9 score (*χ*^*2*^ = 3.76, *p* = .17). The same was found at 12-week follow-up (Week 18) between the LM and the CAU group (*χ*^*2*^ = 2.62, *p* = .32). Despite insignificance upon Fisher’s Exact Test, the LM group had a higher proportion of participants attaining 50% reduction of PHQ-9 score at both time points. Another standard interpretation of significant clinical improvement is a 5-point drop from baseline PHQ-9 score. A significantly higher proportion of participants in the LM group attained a 5-point drop from baseline PHQ-9 score than the CAU group, *χ*^*2*^ = 7.43, *p* = .03, while the group difference narrowed to marginally insignificance at 12-week follow-up (Week 18), *χ*^*2*^ = 5.71, *p* = .057. Taken together, there were some indications of meaningful clinical improvement in the LM group.

### Sensitivity analysis

In order to examine the effects of the LM intervention among the participants with high treatment adherence, only participants (*n* = 6) from the LM group who had completed at least 80% of the sessions (i.e., at least 5 out of 6 sessions) were included in the sensitivity analysis. S4 Table presents the statistical summary of the sensitivity analysis of outcome measures from the baseline to immediate posttreatment and to 12-week follow-up. Linear mixed-effects model analyses demonstrated that PHQ-9 score reduced significantly more in the LM group than the CAU group from the baseline to immediate posttreatment (*p* = .001, *d* = 1.03) and also at 12-week follow-up (*p* = .001, *d* = 1.20) assessment, both with large effect sizes. Moreover, the LM group was also superior to the CAU group in improving DASS–Depression (immediate posttreatment: *p* < .001, *d* = 1.12; 12-week follow-up: *p* < .001, *d* = 0.95), DASS–Anxiety (immediate posttreatment: *p* = .009, *d* = 1.06; 12-week follow-up: *p* = .001, *d* = 0.78), DASS–Stress (immediate posttreatment: *p* = .003, d = 0.59; 12-week follow-up: *p* = .002, *d* = 0.91), SF-6D (immediate posttreatment: *p* = .04, *d* = 0.53; 12-week follow-up: *p* = .006, *d* = 1.14), and SDS (immediate posttreatment: *p* = .03, *d* = 0.81; 12-week follow-up: *p* = .03, *d* = 0.85) from the baseline to immediate posttreatment and 12-week follow-up assessments, with medium to large effect sizes. For improving insomnia symptoms, the LM group was superior to the CAU group in ISI only at 12-week follow up assessment (*p* = .04, *d* = 1.54) but not at immediate posttreatment (*p* = .06). No significant group difference was found in MFI-20 across the time points (*p* = .19 –.29). The clinical significance was examined again, taking into account of the 80% attendance rate using Fisher’s Exact Test. At immediate posttreatment assessment (Week 6), non-significant difference was found between the LM group and the CAU group in the proportion of participants who attained 50% reduction of form baseline PHQ-9 score (*χ*^*2*^ = 2.18, *p* = .32). The same was found at 12-week-follow-up (18 week) between the LM and the CAU group, (*χ*^*2*^ = 3.19, *p* = .28). Using the criteria of 5-point drop from baseline PHQ-9 score, significantly higher proportion of participants in the LM group attained a 5-point drop from baseline PHQ-9 score than the CAU group, *χ*^*2*^ = 6.41, *p* = .04, while the group difference maintained at 12-week follow-up (Week 18), *χ*^*2*^ = 6.27, *p* = .03.

### Treatment credibility, expectancy, and adherence

Paired *t*-tests were performed for the CEQ results in the LM group. Treatment credibility significantly increased from the baseline (*M* = 5.9, *SD* = 1.3) to immediate posttreatment assessment (*M* = 7.3, *SD* = 1.4) in the LM group [*t*(7) = 3.4, *p* = 0.012]. However, no significant difference was found on the treatment expectancy measures (p > .05). Throughout the study, no adverse effects resulting from the LM intervention were reported. Regarding the treatment adherence, out of the 16 participants in the LM group, two of them (12.5%) attended all six treatment sessions, eight of them (50.0%) attended ≥ three sessions, 11 of them (68.8%) attended ≥ one session(s). Five of the participants (31.3%) were absent for all sessions: three of them dropped out from the study before the sessions commenced due to time clash with other activities, the other two missed all the sessions due to physical problems or busy schedules.

### Post-hoc power analysis

The post-hoc power analysis revealed that the power of the present sample size (*n* = 31) in determining the difference in PHQ-9 at immediate posttreatment (*d* = 0.69) and 12-week follow-up (*d* = 0.73) assessments were 45.9% and 50.2% respectively. The effect sizes estimated in this pilot study suggested that a sample size of 74 (37 in each group) and a sample size of 62 (31 in each group) is needed to detect between-group differences in PHQ-9 at immediate posttreatment and 12-week follow-up assessments respectively, with a power level of 80% and a significance level of 0.05.

## Discussion

To the best of our knowledge, this was the first RCT to explore the effects of a truly integrative LM intervention in a group basis on adults with depressive symptoms who were not on current medication or psychological treatment. The present study aimed to examine the effects and acceptability of a group-based, integrative LM intervention in a Chinese (Hong Kong) sample of adults with depressive symptoms, and provided estimates of effect sizes for future fully powered studies. Our findings tentatively suggested that the LM intervention, compared to CAU, could effectively reduce depressive symptoms. The effect sizes at both the end of the intervention and the follow up time point were medium, thereby indicating a notable clinical effect. Using different assessments of clinically significant improvement (50% reduction or 5-point drop from baseline PHQ-9 scores), our findings showed mixed results. For 50% score reduction, there was an insignificant difference in the proportion of participants achieved clinically meaningful improvement between the LM group and the CAU group at both immediate posttreatment (Week 6) and 12-week follow-up assessment (Week 18). In contrast, 5-point drop from baseline PHQ-9 score revealed significantly higher proportions of participants achieving clinical improvements at immediate posttreatment. However, on a cautionary note, the sample size was small (*n* = 31), which limited the interpretation of these findings. Taken into account of the small sample size, we proposed the following explanations. The absence of clinically significant difference using 50% score reduction between the two groups from baseline to immediate posttreatment (Week 6) assessments may be explained by the readiness and commitment to change personal habits. Lifestyle modifications take time to implement and turn into habits or daily routine, thus it is expected that the impacts of lifestyle modifications on depression are likely to occur gradually if an individual persists over a longer period of time. Significant improvements were also found in anxiety and stress symptoms as well as insomnia severity. Encouragingly, no adverse effects resulting from the LM intervention were reported in this study. In line with the meta-analysis and recent RCTs, we showed that LM interventions were superior to CAU in managing depressive symptoms [33–37, 56].

In alignment with results of previous RCTs examining LM interventions for depressed adults, the results of the current study also supported that LM is a safe and effective standalone treatment for depression. Our result (*d* = 0.73 at the 12-week follow-up assessment) was comparable to the 12-week multi-domain lifestyle modification intervention delivered through home visits and telephone calls for depressed older adults, which reported that the LM group resulted in a greater reduction of depressive symptoms among depressed older adults than their counterparts who received usual care (*d* = 0.70) [36]. Although another RCT with four hygienic-dietary recommendations including exercise, diet, sun exposure and sleep as an add-on treatment of depression demonstrated a larger effect size at the 6-month follow-up (*d* = 0.89) [34], the findings could not be replicated by an RCT with a larger sample size and longer follow-up [35]. The researchers suggested that the intervention, in which the lifestyle recommendations were solely delivered in written form and without any monitoring systems of their implementation, could be too limited in motivating depressed patients to accomplish the lifestyle changes. On the other hand, one of the key factors that contributed to the significant improvement in depressive symptoms in our study could be the goal-setting strategies employed in the intervention. Strong evidence indicated that by setting specific and realistic goals, discussing problem-solving skills to remove barriers, and providing some levels of supervision, depressed patients would be more likely to adopt the proposed behavioral changes [57]. Moreover, techniques such as motivational interviewing are likely to be effective in engaging depressed patients to move toward desirable lifestyle changes [58–60]. In the current treatment protocol, we have integrated elements of motivational interviewing and SMART goal-setting in each treatment session. After learning the rationale and practical recommendations for lifestyle modifications, participants were guided to explore their ambivalence to change, learn practical problem-solving skills to overcome potential barriers, and set specific and achievable goals for the coming week. Log sheets were provided to encourage participants to record their daily practice of lifestyle modifications. In the last session, participants were guided to set short-, medium-, and long-term goals for continuous implementation or maintenance of the lifestyle modifications. These goal-setting strategies were believed to continuously motivate and engage our participants in lifestyle modifications throughout the study.

Furthermore, the results of the present study are also in line with a meta-analysis that supported the effectiveness of group therapy for depression (*g* = 0.68) [61]. Researchers have highlighted many advantages of group psychotherapy, such as normalizing one’s experiences through identification with others, positive reinforcement, vicarious learning in a safe environment, and the experience of mastery through suggesting solutions for other group members [62]. Lewinsohn and Clarke (1999) suggested that, by observing others undertaking certain changes, other group members would be more likely to attempt [63]. Our study was conducted in groups, participants were encouraged to participate in interactive group discussions in every treatment session. It was observed that participants were in general willing to share their experience of implementing lifestyle changes in their daily lives and were able to support each other through normalization and exchange of supportive feedback. Thus, one’s success story could positively reinforce behavioral changes in others. We believe these therapeutic factors of group therapy have also contributed to the effects of our group-based LM intervention. Lastly, group-based LM interventions have a huge advantage over individual interventions in terms of cost-effectiveness as they represented only 8–17% of the cost of individual therapies [64].

Regarding the completion rate of the treatment, only 12.5%, 37.5% and 80% of the participants in the LM group achieved 100%, 80% and 50% attendance rate, respectively. Reasons of absence included busy at work or study, time clash with other activities, and feeling physically unwell. It is reflected by some of our participants that as our 6-week treatment overlapped with the university’s examination period, they encountered difficulties in sparing time for the treatment sessions. The venue of the treatment (i.e., the university), which was away from the city centre, could also be one of the barriers for attending treatment sessions. One participant commented that it took much travel time to attend the sessions, which eventually led to her decision to drop out during the treatment. It is recommended that future studies shall take the mode of delivery (such as delivering remotely via online or smartphone app) into considerations in order to facilitate a higher level of adherence. Moreover, it may also encourage treatment adherence by decreasing the duration or the number of treatment sessions.

Given the large variance of attendance rate, a sensitivity analysis was performed to examine the treatment effects among participants who had higher adherence rate (i.e., at least 5 out of 6 treatment sessions). This group of participants showed large effect sizes at the two time points. Effect sizes of anxiety, stress, and insomnia were also larger in this group. Besides, significant improvements in health-related quality of life and functioning were only found in this group. This preliminary result suggested that treatment adherence is crucial in the treatment effects of LM. While a low level of motivation is one of the common symptoms of depression that could hinder treatment adherence and in turn decrease treatment effectiveness, it is suggested that motivational strategies, such as motivational interviewing, goal-setting and behavioral activation, should be considered as standard to encourage participation in future studies.

Lastly, treatment credibility and expectancy were measured and analyzed in the present study. It was found that treatment credibility, as measured by “how believable, convincing, and logical the treatment is”, significantly increased after the intervention. This indicated that the participants in general became more convinced of the treatment rationale and more confident in recommending LM interventions to friends who also experience depressive symptoms. However, no significant changes were found from pre-treatment to post-treatment regarding participants’ subjective beliefs and feelings about the improvements in their functioning through the LM intervention. It indicated that participants’ expectancy on the intervention generally remained unchanged across the treatment.

### Limitations and future directions

Besides the small sample size due to the pilot nature of this trial that required cautious interpretations, there were other limitations in this study. First of all, we opted to use PHQ-9 as a screening tool instead of an independent clinical evaluation by a clinician. The majority of our participants were students, whose depressive symptoms as measured by PHQ-9 could have been induced by stress from temporary study workload and resolved on its own in due time. Therefore, regression to the mean could have occurred that resulted in the detected clinical improvement. The improvement in the CAU group also pointed to this phenomenon. To eliminate this confounding variable, we proposed that future replication studies could consider applying PHQ-9 screening a second time after the initial screening for reliability. Only participants who consistently showed increased PHQ-9 levels would be recruited. With respect to the association of treatment adherence to treatment effects, we did not ask our participants to report their applications of lifestyle modifications (e.g., the amount of time doing exercise or mindfulness practice) in their daily life. While adherence to lifestyle interventions that include behavior changes in daily life can influence the effects of such interventions, it is recommended that common objective measures of adherence, including pedometers and other physiological measurement tools such as heart rate monitors, could be included in future studies to examine the relationship of treatment adherence with treatment effects [65]. The low completion rate and attendance rate in the present study were also one of the limitations. Future studies are advised to incorporate additional motivational strategies and eliminate possible barriers to treatment to enhance treatment adherence. In order to systematically investigate potential factors related to treatment adherence and reasons for dropout, independent assessments by third party or focus group discussions may be considered in future studies. Given that lifestyle modifications take time to integrate into daily routine naturally, and their impacts on depressive symptoms through complex physiological mechanisms could also take time to realize, it would be of interest to provide a potentially longer (flexible) intervention, with a longer follow up period and multiple time points to examine the sustained effects of LM modifications for the management of depression.

## Conclusion

In conclusion, to our best knowledge, our study was the first pilot RCT to examine the effects of integrated LM intervention in managing depressive symptoms in the Chinese population. We demonstrated that the group-based, integrated LM intervention could be an effective and acceptable treatment option for depressed adults. Further evaluation of fully powered RCTs is warranted to evaluate the robustness of LM interventions for depression (potentially via an online or smartphone app delivery). With further support from future studies, LM interventions could well be considered as a safe and cost-effective treatment option for the prevention, management, and treatment of depression in community mental health services.

## Supporting information

S1 Checklist CONSORT 2010 checklist of information to include when reporting a randomised trial*. (DOC)

S1 Table Treatment overview. (PDF)

S2 Table Demographics and baseline measures. Values are expressed in means ± standard deviations or n (%); LM, Lifestyle Medicine; CAU, Care as Usual; PHQ-9, Patient Health Questionnaire; DASS, Depression Anxiety Stress Scales; ISI, Insomnia Severity Index; MFI, Multidimensional Fatigue Inventory; SF-6D, Short-Form 6-Dimension; SDS, Sheehan Disability Scale. Data are presented as mean ± standard deviation or number (%). Independent *t*-test or Fisher’s exact test were used for comparisons. ^†^ Self-report lifetime history of clinical diagnosis.
(PDF)

S3 Table Effects of LM intervention at the immediate posttreatment (Week 6) and 12-week follow-up (Week 18) assessments. PHQ-9, Patient Health Questionnaire; DASS, Depression Anxiety Stress Scales; ISI, Insomnia Severity Index; MFI, Multidimensional Fatigue Inventory; SF-6D, Short-Form 6-Dimension; SDS, Sheehan Disability Scale. ^†^ Mixed-effects models group by time interaction. Immediate posttreatment: 2 (groups) x 2 (time points); 12-week follow-up: 2 (groups) x 3 (time points).
(PDF)

S4 Table Sensitivity analysis of the effects of LM intervention at the immediate posttreatment (Week 6) and 12-week follow-up (Week 18) assessments. Values are expressed in means ± standard deviations; LM = Lifestyle Medicine; CAU = Care as Usual; PHQ-9, Patient Health Questionnaire; DASS, Depression Anxiety Stress Scales; ISI, Insomnia Severity Index; MFI, Multidimensional Fatigue Inventory; SF-6D, Short-Form 6-Dimension; SDS, Sheehan Disability Scale. ^†^ Mixed-effects models group by time interaction. Immediate posttreatment: 2 (groups) x 2 (time points); 12-week follow-up: 2 (groups) x 3 (time points).
(PDF)

S1 Data (DOCX)

S1 File (PDF)

## References

[1] American Psychiatric Association. Diagnostic and statistical manual of mental disorders, fifth edition. Arlington, VA: American Psychiatric Association; 2013.

[2] Woo JM, Jeon HJ, Noh E, Kim HJ, Lee SW, Lee K, et al. Importance of remission and residual somatic symptoms in health-related quality of life among outpatients with major depressive disorder: a cross-sectional study. Health and Quality of Life Outcomes. 2014;12(188). doi: 10.1186/s12955-014-0188-y 25519704 PMC4280041

[3] World Health Organization. Depression and other common mental disorders: global health estimates. Geneva: World Health Organization; 2017:1–24.

[4] Ni MY, Yao XI, Leung KSM, Yau C, Leung CMC, Lun P, et al. Depression and post-traumatic stress during major social unrest in Hong Kong: a 10-year prospective cohort study. Lancet. 2020; 395(10220): 273–284. doi: 10.1016/S0140-6736(19)33160-5 31928765

[5] World Health Organization. Public health action for the prevention of suicide: a framework. 2012. Available from: https://apps.who.int/iris/bitstream/handle/10665/75166/9789241503570_eng.pdf

[6] Mathers CD, Loncar D. Projections of global mortality and burden of disease from 2002 to 2030. PLoS Med. 2006; 3(11): e442. doi: 10.1371/journal.pmed.0030442 17132052 PMC1664601

[7] Gall SL, Sanderson K, Smith KJ, Patton G, Dwyer T, Venn A. Bi-directional associations between healthy lifestyles and mood disorders in young adults: the childhood determinants of adult health study. Psychol Med. 2016; 46(12): 2535–2548. doi: 10.1017/S0033291716000738 27338017

[8] Velten J, Lavallee KL, Scholten S, Meyer AH, Zhang XC, Schneider S, et al. Lifestyle choices and mental health: a representative population survey. BMC Psychol. 2014;2(1):58. doi: 10.1186/s40359-014-0055-y 25628891 PMC4304169

[9] Guthrie G. Definition of lifestyle medicine. In: Ripple JM. Lifestyle Medicine. Boca Raton: CRC Press; 2019: pp. 961–968.

[10] Dunkley AJ, Charles K, Gray LJ, Camosso-Stefinovic J, Davies MJ, Khunti K. Effectiveness of interventions for reducing diabetes and cardiovascular disease risk in people with metabolic syndrome: systematic review and mixed treatment comparison meta-analysis. Diabetes Obes Metab. 2012; 14(7): 616–625. doi: 10.1111/j.1463-1326.2012.01571.x 22284386

[11] Morton DP. Combining lifestyle medicine and positive psychology to improve mental health and emotional well-being. Am J Lifestyle Med. 2018; 12(5): 370–374. doi: 10.1177/1559827618766482 30283261 PMC6146362

[12] Berk M, Williams LJ, Jacka FN, O’Neil A, Pasco JA, Moylan S, et al. So depression is an inflammatory disease, but where does the inflammation come from? BMC Med. 2013; 11: 200. doi: 10.1186/1741-7015-11-200 24228900 PMC3846682

[13] Lopresti AL, Hood SD, Drummond PD. A review of lifestyle factors that contribute to important pathways associated with major depression: diet, sleep and exercise. J Affect Disord. 2013; 148(1): 12–27. doi: 10.1016/j.jad.2013.01.014 23415826

[14] Sarris J, O’Neil A, Coulson CE, Schweitzer I, Berk M. Lifestyle medicine for depression. BMC Psychiatry. 2014; 14: 107. doi: 10.1186/1471-244X-14-107 24721040 PMC3998225

[15] Walsh R. Lifestyle and mental health. Am Psychol. 2011; 66(7): 579–592. doi: 10.1037/a0021769 21244124

[16] Berk M, Sarris J, Coulson CE, Jacka FN. Lifestyle management of unipolar depression. Acta Psychiatr Scand Suppl. 2013; 127(443): 38–54. 10.1111/acps.12124 23586875

[17] Lai JS, Hiles S, Bisquera A, Hure AJ, McEvoy M, Attia J. A systematic review and meta-analysis of dietary patterns and depression in community-dwelling adults. Am J Clin Nutr. 2014; 99(1): 181–197. doi: 10.3945/ajcn.113.069880 24196402

[18] Lassale C, Batty GD, Baghdadli A, et al. Correction: Healthy dietary indices and risk of depressive outcomes: a systematic review and meta-analysis of observational studies. Mol Psychiatry. 2019; 24: 965–986. doi: 10.1038/s41380-018-0237-8 30254236 PMC6755986

[19] Molendijk M, Molero P, Ortuño Sánchez-Pedreño F, Van der Does W, Angel Martínez-González M. Diet quality and depression risk: a systematic review and dose-response meta-analysis of prospective studies. J Affect Disord. 2018; 226: 346–354. doi: 10.1016/j.jad.2017.09.022 29031185

[20] Jacka FN, O’Neil A, Opie R, Itsiopoulos C, Cotton S, Mohebbi M, et al. A randomised controlled trial of dietary improvement for adults with major depression (the “SMILES” trial). BMC Med. 2017;15(1): 23. doi: 10.1186/s12916-017-0791-y 28137247 PMC5282719

[21] Firth J, Marx W, Dash S, Carney R, Teasdale SB, Solmi M, et al. The effects of dietary improvement on symptoms of depression and anxiety: a meta-analysis of randomized controlled trials. Psychosom Med. 2019; 81(3): 265–280. doi: 10.1097/PSY.0000000000000673 30720698 PMC6455094

[22] Kvam S, Kleppe CL, Nordhus IH, Hovland A. Exercise as a treatment for depression: a meta-analysis. J Affect Disord. 2016; 202: 67–86. doi: 10.1016/j.jad.2016.03.063 27253219

[23] Rebar AL, Stanton R, Geard D, Short C, Duncan MJ, Vandelanotte C. A meta-meta-analysis of the effect of physical activity on depression and anxiety in non-clinical adult populations. Health Psychol Rev. 2015; 9(3): 366–378. doi: 10.1080/17437199.2015.1022901 25739893

[24] Schuch FB, Vancampfort D, Richards J, Rosenbaum S, Ward PB, Stubbs B. Exercise as a treatment for depression: a meta-analysis adjusting for publication bias. J Psychiatr Res. 2016; 77: 42–51. doi: 10.1016/j.jpsychires.2016.02.023 26978184

[25] Ashdown-Franks G, Firth J, Carney R, et al. Exercise as medicine for mental and substance use disorders: a meta-review of the benefits for neuropsychiatric and cognitive outcomes. Sports Med. 2020; 50(1): 151–170. doi: 10.1007/s40279-019-01187-6 31541410

[26] Carek PJ, Laibstain SE, Carek SM, Carvalho AF, Hallgren M, Koyanagi A, et al. Exercise for the treatment of depression and anxiety. Int J Psychiatry Med. 2011; 41(1): 15–28. doi: 10.2190/PM.41.1.c 21495519

[27] Cooney G, Dwan K, Mead G. Exercise for depression. JAMA. 2014; 311(23): 2432. doi: 10.1001/jama.2014.4930 24938566

[28] Rimer J, Dwan K, Lawlor DA, Greig CA, McMurdo M, Morley W, et al. Exercise for depression. Cochrane Database Syst Rev. 2012;7: CD004366. doi: 10.1002/14651858.CD004366.pub5 22786489

[29] Staner L. Comorbidity of insomnia and depression. Sleep Med Rev. 2010;14(1): 35–46. doi: 10.1016/j.smrv.2009.09.003 19939713

[30] Riemann D, Perlis ML. The treatments of chronic insomnia: a review of benzodiazepine receptor agonists and psychological and behavioral therapies. Sleep Med Rev. 2009;13(3): 205–214. doi: 10.1016/j.smrv.2008.06.001 19201632

[31] Wang YY, Li XH, Zheng W, Xu ZY, Ng CH, Ungvari GS, et al. Mindfulness-based interventions for major depressive disorder: a comprehensive meta-analysis of randomized controlled trials. J Affect Disord. 2018;229: 429–436. doi: 10.1016/j.jad.2017.12.093 29331704

[32] Goldberg SB, Tucker RP, Greene PA, Davidson RJ, Wampold BE, Kearney DJ, et al. Mindfulness-based interventions for psychiatric disorders: a systematic review and meta-analysis. Clin Psychol Rev. 2018;59: 52–60. doi: 10.1016/j.cpr.2017.10.011 29126747 PMC5741505

[33] Wong VW, Ho FY, Shi NK, Sarris J, Chung KF, Yeung WF. Lifestyle medicine for depression: a meta-analysis of randomized controlled trials. J Affect Disord. 2021;284:203–216. doi: 10.1016/j.jad.2021.02.012 33609955

[34] García-Toro M, Ibarra O, Gili M, Serrano MJ, Oliván B, Vicens E, et al. Four hygienic-dietary recommendations as add-on treatment in depression: a randomized-controlled trial. J Affect Disord. 2012;140(2): 200–203. doi: 10.1016/j.jad.2012.03.031 22516309

[35] Serrano Ripoll MJ, Oliván-Blázquez B, Vicens-Pons E, Roca M, Gili M, Leiva A, et al. Lifestyle change recommendations in major depression: do they work? J Affect Disord. 2015;183: 221–228. doi: 10.1016/j.jad.2015.04.059 26025368

[36] Chang KJ, Hong CH, Roh HW, Lee KS, Lee EH, Kim J, et al. A 12-week multi-domain lifestyle modification to reduce depressive symptoms in older adults: a preliminary report. Psychiatry Investig. 2018;15(3): 279–284. doi: 10.30773/pi.2017.08.10 29475242 PMC5900365

[37] Roh HW, Hong CH, Lim HK, Chang KJ, Kim H, Kim NR, et al. A 12-week multidomain intervention for late-life depression: a community-based randomized controlled trial. J Affect Disord. 2020;263: 437–444. doi: 10.1016/j.jad.2019.12.013 31969275

[38] Murphy JA, Oliver G, Ng CH, Wain C, Magennis J, Opie RS, et al. Pilot-testing of “Healthy Body Healthy Mind”: an integrative lifestyle program for patients with a mental illness and co-morbid metabolic syndrome. Front Psychiatry. 2019;10: 91. doi: 10.3389/fpsyt.2019.00091 30894821 PMC6414430

[39] Kroenke K, Spitzer RL, Williams JB. The PHQ‐9: validity of a brief depression severity measure. J Gen Intern Med. 2001;16(9): 606–613. doi: 10.1046/j.1525-1497.2001.016009606.x 11556941 PMC1495268

[40] Spitzer RL, Kroenke K, Williams JB. Validation and utility of a self-report version of PRIME-MD: the PHQ primary care study. JAMA. 1999; 282(18): 1737–1744. doi: 10.1001/jama.282.18.1737 10568646

[41] Yu X, Tam WWS, Wong PTK, Lam TH, Stewart SM. The Patient Health Questionnaire-9 for measuring depressive symptoms among the general population in Hong Kong. Compr Psychiatry. 2012;53(1): 95–102. doi: 10.1016/j.comppsych.2010.11.002 21193179

[42] Lovibond SH, Lovibond PF. Manual for the Depression Anxiety Stress Scales. Psychology Foundation of Australia; 1995.

[43] Lovibond PF, Lovibond SH. The structure of negative emotional states: comparison of the Depression Anxiety Stress Scales (DASS) with the Beck Depression and Anxiety Inventories. 1995;33(3):335–343. doi: 10.1016/0005-7967(94)00075-u 7726811

[44] Taouk M, Lovibond PF, Laube R. Psychometric properties of a Chinese version of the short Depression Anxiety Stress Scales (DASS21). Sydney: Cumberland Hospital; 2001.

[45] Bastien CH, Vallières A, Morin CM. Validation of the Insomnia Severity Index as an outcome measure for insomnia research. Sleep Med. 2001;2(4):297–307. doi: 10.1016/s1389-9457(00)00065-4 11438246

[46] Chung KF, Kan KKK, Yeung WF. Assessing insomnia in adolescents: comparison of Insomnia Severity Index, Athens Insomnia Scale and Sleep Quality Index. Sleep Med. 2011;12(5): 463–470. doi: 10.1016/j.sleep.2010.09.019 21493134

[47] Brazier J, Roberts J, Deverill M. The estimation of a preference-based measure of health from the SF-36. J Health Econ. 2002;21(2): 271–292. doi: 10.1016/s0167-6296(01)00130-8 11939242

[48] McGhee SM, Brazier J, Lam CLK, Wong LC, Chau J, Cheung A, et al. Quality-adjusted life years: population-specific measurement of the quality component. Hong Kong Med J. 2011;17 (Suppl 6):17–21. 22147354

[49] Lam CLK, Brazier J, McGhee SM. Valuation of the SF-6D health states is feasible, acceptable, reliable, and valid in a Chinese population. Value Health. 2008;11(2): 295–303. doi: 10.1111/j.1524-4733.2007.00233.x 18380642

[50] Smets EM, Garssen B, Bonke B, De Haes JC. The Multidimensional Fatigue Inventory (MFI) psychometric qualities of an instrument to assess fatigue. J Psychosom Res. 1995;39(3): 315–325. doi: 10.1016/0022-3999(94)00125-o 7636775

[51] Chung KF, Yu BYM, Yung KP, Yeung WF, Ng TH, Ho FYY. Assessment of fatigue using the Multidimensional Fatigue Inventory in patients with major depressive disorder. Compr Psychiatry. 2014;55(7):1671–1678. doi: 10.1016/j.comppsych.2014.06.006 25035160

[52] Sheehan DV. The Anxiety Disease. New York: Bantam Books; 1983.

[53] Zeljic K, Zhang Y, Qiu X, Chen X, Gong H, Jin H, et al. An evaluation of the psychometric properties of the Sheehan Disability Scale in a Chinese psychotherapy-seeking sample. J Cogn Psychother. 2020;34(1):58–69. doi: 10.1891/0889-8391.34.1.58 32701476

[54] Devilly GJ, Borkovec TD. Psychometric properties of the credibility/expectancy questionnaire. J Behav Ther Exp Psychiatry. 2000;31(2):73–86. doi: 10.1016/s0005-7916(00)00012-4 11132119

[55] Julious SA. Sample size of 12 per group rule of thumb for a pilot study. Pharmaceut Statist. 2005:287–291.

[56] Gómez-Gómez I, Bellón JÁ, Resurrección DM, Cuijpers P, Moreno-Peral P, Rigabert A, et al. Effectiveness of universal multiple-risk lifestyle interventions in reducing depressive symptoms: systematic review and meta-analysis. Prev Med. 2020;134:106067. doi: 10.1016/j.ypmed.2020.106067 32194097

[57] Stanton R, Reaburn P. Exercise and the treatment of depression: a review of the exercise program variables. J Sci Med Sport. 2014;17(2):177–182. doi: 10.1016/j.jsams.2013.03.010 23602562

[58] Hettema J, Steele J, Miller WR. Motivational interviewing. Annu Rev Clin Psychol. 2005;1(1):91–111. doi: 10.1146/annurev.clinpsy.1.102803.143833 17716083

[59] Tahan HA, Sminkey PV. Motivational interviewing: building rapport with clients to encourage desirable behavioural and lifestyle changes. Prof case manag. 2012:17(4), 164–172. doi: 10.1097/NCM.0b013e318253f029 22660338

[60] Westra HA. Managing resistance in cognitive behavioural therapy: the application of motivational interviewing in mixed anxiety and depression. Cogn Behav Ther. 2004;33(4):161–175. doi: 10.1080/16506070410026426 15625790

[61] Okumura Y, Ichikura K. Efficacy and acceptability of group cognitive behavioral therapy for depression: a systematic review and meta-analysis. J Affect Disord. 2014;164:155–164. doi: 10.1016/j.jad.2014.04.023 24856569

[62] Whitfield G. Group cognitive–behavioural therapy for anxiety and depression. Adv Psychiatr Treat. 2010;16(3):219–227.

[63] Lewinsohn PM, Clarke GN. Psychosocial treatments for adolescent depression. Clin Psychol Rev. 1999;19(3):329–342. doi: 10.1016/s0272-7358(98)00055-5 10097874

[64] Peterson AL, Halstead TS. Group cognitive behavior therapy for depression in a community setting: a clinical replication series. Behav Ther. 1998;29(1):3–18.

[65] Nagpal TS, Prapavessis H, Campbell C, Mottola MF. Measuring adherence to a nutrition and exercise lifestyle intervention: is program adherence related to excessive gestational weight gain?. Behav Anal Pract. 2017;10(4):347–54. doi: 10.1007/s40617-017-0189-5 29214130 PMC5711739

