# Supplementary material for: Effects of a group-based lifestyle medicine for depression: A pilot randomized controlled trial
Source: PLoS One. 2021 Oct 8;16(10):e0258059. doi: 10.1371/journal.pone.0258059 (PMC8500430; doi:10.1371/journal.pone.0258059)
Supplement: S1 Table — (PDF) [file pone.0258059.s002.pdf]

S1 Table. Treatment Overview.

Session	Treatment Components	Contents of Lifestyle Intervention
1	Psychoeducation	Integrated health model, stress responses
	Mindfulness	Introduction, breathing space
	Goal-setting	Commitment to program
2	Sleep	Stimulus control, sleep hygiene, progressive muscle relaxation
	Mindfulness	Mindfulness & sleep
	Goal-setting	Strategies for change
3	Psychoeducation	Myths & benefits of physical exercise
	Exercise	Exercise instructions
	Goal-setting	SMART goal-setting
4	Diet	Mood and food
		Dietary guidelines (from both Western and Chinese medicine perspectives)
	Goal-setting	Dealing with set-backs
5	Diet	Dietary guidelines (Glycemic index, probiotics, food label, inflammation, and antioxidants)
	Mindfulness	Emotional eating and mindful eating
	Goal-setting	SMART goal-setting revisit
6	Psychoeducation	Green space
	Mindfulness	Self-compassion
	Goal-setting	Roundup review, goal setting (short-, medium-, and long-term)
