# Supplementary material for: Effects of a group-based lifestyle medicine for depression: A pilot randomized controlled trial
Source: PLoS One. 2021 Oct 8;16(10):e0258059. doi: 10.1371/journal.pone.0258059 (PMC8500430; doi:10.1371/journal.pone.0258059)
Supplement: S2 Table — Values are expressed in means ± standard deviations or n (%); LM, Lifestyle Medicine; CAU, Care as Usual; PHQ-9, Patient Health Questionnaire; DASS, Depression Anxiety Stress Scales; ISI, Insomnia Severity Index; MFI, Multidimensional Fatigue Inventory; SF-6D, Short-Form 6-Dimension; SDS, Sheehan Disability Scale. Data are presented as mean ± standard deviation or number (%). Independent t-test or Fisher’s exact test were used for comparisons. † Self-report lifetime history of clinical diagnosis. (PDF) [file pone.0258059.s003.pdf]

S2 Table *Demographics and baseline measures*

| Measure | LM Group ($n$ = 16) | CAU Group ($n$ = 15) | Total ($n$ = 31) |
|---|---|---|---|
| Age, year | 34.3 ± 15.0 | 35.8 ± 16.4 | 35.1 ± 15.4 |
| Sex, male / female | 4 / 12 | 1 / 14 | 5 / 26 |
| Marital status | | | |
| Single | 10 (62.5) | 10 (66.7) | 20 (64.5) |
| Married | 5 (31.3) | 3 (20) | 8 (25.8) |
| Divorced | 1 (6.3) | 2 (13.3) | 3 (9.7) |
| Education Level | | | |
| None | 2 (12.5) | 0 (0) | 2 (6.5) |
| Primary school | 0 (0) | 0 (0) | 0 (0) |
| Secondary school | 3 (18.8) | 5 (33.3) | 8 (25.8) |
| University or above | 11 (68.8) | 10 (66.7) | 21 (67.7) |
| Occupation | | | |
| Executives or professionals | 5 (31.3) | 0 (0) | 5 (16.1) |
| Clerical or production workers | 4 (25.0) | 5 (33.3) | 9 (29.0) |
| Students | 5 (31.3) | 7 (46.7) | 12 (38.7) |
| Housemakers | 1 (6.3) | 0 (0) | 1 (3.2) |
| Retired | 0 (0) | 1 (6.7) | 1 (3.2) |
| Unemployed/others | 1 (6.3) | 2 (13.3) | 3 (9.7) |
| Previous professional help | | | |
| Any | 4 (25) | 1 (6.7) | 5 (16.1) |
| Psychiatrists | 1 (6.3) | 0 (0) | 1 (3.2) |
| GP or other doctors | 0 (0) | 0 (0) | 0 (0) |
| TCM practitioners | 0 (0) | 0 (0) | 0 (0) |
| Clinical psychologists | 0 (0) | 0 (0) | 0 (0) |
| Counsellors, nurses or others | 3 (18.8) | 1 (6.7) | 4 (12.9) |
| Previous treatment of depression | | | |
| Any | 3 (18.8) | 3 (20) | 6 (19.4) |
| Prescribed medications | 1 (6.3) | 0 (0) | 1 (3.2) |
| Traditional Chinese Medicine | 1 (6.3) | 2 (13.4) | 3 (9.7) |
| Acupuncture, acupressure, or reflexology | 1 (6.3) | 1 (6.7) | 2 (6.5) |
| Alcohol use ≥1 time/wk | 3 (18.8) | 2 (13.3) | 5 (16.1) |
| Smoking ≥1 time/wk | 1 (6.3) | 0 (0) | 1 (3.2) |
| Physical activities ≥1 time/wk | 7 (43.8) | 5 (33.3) | 12 (38.7) |
| Mindfulness practice ≥1 time/wk | 2 (12.5) | 2 (13.3) | 4 (12.9) |
| Psychiatric disorders[†] | | | |
| Any | 2 (12.5) | 3 (20.0) | 5 (16.1) |
| Depressive disorder | 2 (12.5) | 3 (20.0) | 5 (16.1) |
| Others | 0 (0) | 0 (0) | 0 (0) |

| Measure | LM Group ($n$ = 16) | CAU Group ($n$ = 15) | Total ($n$ = 31) |
|---|---|---|---|
| Physical disorders [†] | | | |
| Any | 5 (31.3) | 5 (33.3) | 10 (32.3) |
| Cancer | 1 (6.3) | 0 (0) | 1 (3.2) |
| Pain disorder | 1 (6.3) | 2 (13.3) | 3 (9.7) |
| Respiratory disease | 1 (6.3) | 1 (6.7) | 2 (6.5) |
| Diabetes or other endocrine disorder | 0 (0) | 1 (6.7) | 1 (3.2) |
| Others | 2 (12.5) | 1 (6.7) | 3 (9.7) |
| Baseline outcome measures | | | |
| PHQ-9 | 13.5 ± 3.0 | 11.5 ± 3.0 | 12.6 ± 3.1 |
| DASS – Depression | 18.9 ± 7.5 | 15.2 ± 7.6 | 17.1 ± 7.6 |
| DASS – Anxiety | 14.1 ± 8.7 | 12.7 ± 5.5 | 13.4 ± 7.2 |
| DASS – Stress | 23.3 ± 9.3 | 18.4 ± 7.3 | 20.9 ± 8.6 |
| ISI | 13.5 ± 5.2 | 11.4 ± 6.6 | 12.5 ± 5.9 |
| MFI-20 | 62.3 ± 4.3 | 63.9 ± 5.3 | 63.1 ± 4.8 |
| SF-6D | 0.6 ± 0.08 | 0.6 ± 0.09 | 0.6 ± 0.08 |
| SDS | 9.8 ± 8.3 | 9.5 ± 7.4 | 9.7 ± 7.8 |

Values are expressed in means ± standard deviations or *n* (%); LM, Lifestyle Medicine; CAU, Care as Usual; PHQ-9, Patient Health Questionnaire; DASS, Depression Anxiety Stress Scales; ISI, Insomnia Severity Index; MFI, Multidimensional Fatigue Inventory; SF-6D, Short-Form 6-Dimension; SDS, Sheehan Disability Scale.

Data are presented as mean ± standard deviation or number (%).

Independent *t*-test or Fisher's exact test were used for comparisons.

[†] Self-report lifetime history of clinical diagnosis
