# Supplementary material for: Effects of a group-based lifestyle medicine for depression: A pilot randomized controlled trial
Source: PLoS One. 2021 Oct 8;16(10):e0258059. doi: 10.1371/journal.pone.0258059 (PMC8500430; doi:10.1371/journal.pone.0258059)
Supplement: S3 Table — PHQ-9, Patient Health Questionnaire; DASS, Depression Anxiety Stress Scales; ISI, Insomnia Severity Index; MFI, Multidimensional Fatigue Inventory; SF-6D, Short-Form 6-Dimension; SDS, Sheehan Disability Scale. † Mixed-effects models group by time interaction. Immediate posttreatment: 2 (groups) x 2 (time points); 12-week follow-up: 2 (groups) x 3 (time points). (PDF) [file pone.0258059.s004.pdf]

S3 Table. Effects of LM Intervention at the immediate posttreatment (Week 6) and 12-week follow-up (Week 18) assessments.

Measure	LM Group (n = 16)	CAU Group (n = 15)		
	Mean ± SD	Mean ± SD	Between-group effect size (95% CI)	p-value †
PHQ-9				
Baseline	13.5 ± 3.0	11.5 ± 3.0		
Immediate posttreatment	7.4 ± 2.3	9.5 ± 3.7	-0.69 (-1.39, 0.05)	0.02
12-week follow-up	7.5 ± 3.6	10.2 ±3.8	-0.73 (-1.44, 0.02)	0.01
DASS – Depression				
Baseline	18.9 ± 7.5	15.2 ± 7.6		
Immediate posttreatment	7.0 ± 3.7	12.9 ± 7.8	-0.98 (-1.69, -0.21)	0.008
12-week follow-up	9.5 ± 7.8	13.3 ± 9.0	-0.45 (-1.15, 0.27)	0.008
DASS – Anxiety				
Baseline	14.1 ± 8.7	12.7 ± 5.5		
Immediate posttreatment	4.3 ± 2.5	11.1 ± 8.0	-1.16 (-1.89, -0.37)	0.03
12-week follow-up	6.5 ± 2.8	10.7 ± 7.3	-0.77 (-1.48, -0.02)	0.03
DASS – Stress				
Baseline	23.3 ± 9.3	18.4 ± 7.3		
Immediate posttreatment	13.5 ± 7.7	17.1 ± 9.6	-0.42 (-1.12, 0.31)	0.04
12-week follow-up	11.5 ± 6.9	16.9 ± 9.6	-0.65 (-1.35, 0.09)	0.04
ISI				
Baseline	13.5 ± 5.2	11.4 ± 6.6		
Immediate posttreatment	7.6 ± 3.6	11.6 ± 6.4	-0.78 (-1.49, -0.03)	0.048
12-week follow-up	6.4 ± 4.8	10.7 ± 4.4	-0.93 (-1.65, -0.17)	0.046
MFI-20				
Baseline	62.3 ± 4.3	63.9 ± 5.3		
Immediate posttreatment	65.6 ± 6.8	62.9 ± 9.4	0.33 (-0.39, 1.03)	0.21
12-week follow-up	65.5 ± 5.7	63.5 ± 6.5	0.33 (-0.39, 1.03)	0.35
SF-6D				
Baseline	0.61 ± 0.08	0.63 ± 0.09		
Immediate posttreatment	0.70 ± 0.10	0.66 ± 0.11	0.38 (-0.34, 1.08)	0.12
12-week follow-up	0.74 ± 0.17	0.68 ± 0.14	0.38 (0.34, 1.09)	0.29
SDS				
Baseline	9.8 ± 8.3	9.5 ± 7.4		
Immediate posttreatment	3.6 ± 3.1	10.1 ± 8.9	-0.99 (-1.71, -0.22)	0.09
12-week follow-up	2.1 ± 2.6	7.9 ± 8.2	-0.97 (-1.68, -0.20)	0.13

Values are expressed in means ± standard deviations; LM, Lifestyle Medicine; CAU,Care as Usual; PHQ-9, Patient Health Questionnaire; DASS, Depression Anxiety Stress Scales; ISI,

Insomnia Severity Index; MFI, Multidimensional Fatigue Inventory; SF-6D, Short-Form 6-Dimension; SDS, Sheehan Disability Scale.
† Mixed-effects models group by time interaction. Immediate posttreatment: 2 (groups) x 2 (time points); 12-week follow-up: 2 (groups) x 3 (time points)
