# Supplementary material for: Effects of a group-based lifestyle medicine for depression: A pilot randomized controlled trial
Source: PLoS One. 2021 Oct 8;16(10):e0258059. doi: 10.1371/journal.pone.0258059 (PMC8500430; doi:10.1371/journal.pone.0258059)
Supplement: S4 Table — Values are expressed in means ± standard deviations; LM = Lifestyle Medicine; CAU = Care as Usual; PHQ-9, Patient Health Questionnaire; DASS, Depression Anxiety Stress Scales; ISI, Insomnia Severity Index; MFI, Multidimensional Fatigue Inventory; SF-6D, Short-Form 6-Dimension; SDS, Sheehan Disability Scale. † Mixed-effects models group by time interaction. Immediate posttreatment: 2 (groups) x 2 (time points); 12-week follow-up: 2 (groups) x 3 (time points). (PDF) [file pone.0258059.s005.pdf]

S4 Table. Sensitivity analysis of the effects of LM Intervention at the immediate posttreatment (Week 6) and 12-week follow-up (Week 18) assessments.

Measure	LM Group (n = 6)	CAU Group (n = 15)		
	Mean ± SD	Mean ± SD	Between-group effect size (95% CI)	p-value †
PHQ-9				
Baseline	14.2 ± 3.0	11.5 ± 3.0		
Immediate posttreatment	6.2 ± 0.8	9.5 ± 3.7	-1.03 (-1.98, 0.00)	0.001
12-week follow-up	6.0 ±2.4	10.2 ±3.8	-1.20 (-2.17, -0.15)	0.001
DASS – Depression				
Baseline	21.7 ± 6.1	15.2 ± 7.6		
Immediate posttreatment	5.3 ± 2.1	12.9 ± 7.8	-1.12 (-2.08, -0.07)	< 0.001
12-week follow-up	5.7 ± 3.9	13.3 ± 9.0	-0.95 (-1.90, 0.07)	< 0.001
DASS – Anxiety				
Baseline	16.3 ± 8.6	12.7 ± 5.5		
Immediate posttreatment	3.7 ± 2.3	11.1 ± 8.0	-1.06 (-2.02, -0.02)	0.009
12-week follow-up	5.7 ±2.7	10.7 ± 7.3	-0.78 (-1.72, 0.23)	0.004
DASS – Stress				
Baseline	26.7 ± 9.1	18.4 ± 7.3		
Immediate posttreatment	11.7 ± 7.5	17.1 ± 9.6	-0.59 (-1.53, 0.39)	0.003
12-week follow-up	9.0 ± 5.2	16.9 ± 9.6	-0.91 (-1.86, 0.11)	0.002
ISI				
Baseline	12.2 ± 7.2	11.4 ± 6.6		
Immediate posttreatment	6.2 ± 2.7	11.6 ± 6.4	-0.95 (-1.90, 0.07)	0.060
12-week follow-up	4.3 ± 3.4	10.7 ± 4.4	-1.54 (-2.52, -0.43)	0.043
MFI-20				
Baseline	62.7 ± 3.9	63.9 ± 5.3		
Immediate posttreatment	66.9 ± 7.3	62.9 ± 9.4	0.45 (-0.52, 1.39)	0.188
12-week follow-up	66.7 ±6.2	63.5 ± 6.5	0.50 (-0.48, 1.44)	0.294
SF-6D				
Baseline	0.59 ± 0.08	0.63 ± 0.09		
Immediate posttreatment	0.72 ± 0.12	0.66 ± 0.11	0.53 (-0.45, 1.47)	0.039
12-week follow-up	0.82 ± 0.05	0.68 ± 0.14	1.14 (0.09, 2.10)	0.006
SDS				
Baseline	13.0 ± 8.9	9.5 ± 7.4		
Immediate posttreatment	3.8 ± 3.1	10.1 ± 8.9	-0.81 (-1.75, 0.20)	0.032
12-week follow-up	1.8 ± 2.4	7.9 ± 8.2	-0.85 (-1.80, 0.16)	0.028

Values are expressed in means ± standard deviations; LM= Lifestyle Medicine; CAU= Care as Usual; PHQ-9, Patient Health Questionnaire; DASS, Depression Anxiety Stress Scales; ISI, Insomnia Severity Index; MFI, Multidimensional Fatigue Inventory; SF-6D, Short-Form 6-Dimension; SDS, Sheehan Disability Scale.

† Mixed-effects models group by time interaction. Immediate posttreatment: 2 (groups) x 2 (time points); 12-week follow-up: 2 (groups) x 3 (time points)
