# Supplementary material for: Effects of a group-based lifestyle medicine for depression: A pilot randomized controlled trial
Source: PLoS One. 2021 Oct 8;16(10):e0258059. doi: 10.1371/journal.pone.0258059 (PMC8500430; doi:10.1371/journal.pone.0258059)
Supplement: S1 Data — (DOCX) [file pone.0258059.s006.docx]

**List of Instruments**

The following self-report measures will be used.

*Primary outcome measure*

1. **The Patient Health Questionnaire (PHQ-9; Kroenke, Spitzer, & Williams, 2001)**

The PHQ-9, a 20-item questionnaire used for screening, diagnosing, monitoring and measuring the severity of depression, which scores each of the nine DSM-IV criteria as “0” (not at all) to “3” (nearly every day).

*Secondary outcome measures*

1. **Depression Anxiety Stress Scales (DASS-21; Lovibond & Lovibond, 1995)**

DASS-21 is a 21-items scales, comprises three sub-scales which measures the negative emotional states of depression, anxiety, and stress, over the past week. The DASS is based on a dimensional rather than a categorical conception of psychological disorder, thus it has no direct implications for the allocation of patients to discrete diagnostic categories. However, recommended cutoffs for conventional severity labels (normal, moderate, severe) are given in the DASS Manual.

1. **Insomnia Severity Index (ISI; Bastien, Vallieres, & Morin, 2001)**

ISI is a 7-item scale designed to evaluate perceived insomnia severity. Ratings on the 5-point Likert scale are obtained on the perceived severity of sleep-onset, sleep-maintenance, early morning awakening problems, satisfaction with current sleep pattern, interference with daily functioning, noticeably of impairment attributed to the sleep problem, and level of distress caused by the sleep problem.

1. **Short form Health Survey – 36 items (SF-36; Ware, Kosinski, Dewey, & Gandek, 1993)**

SF-36 is used to assess the patients’ general health and quality of life. It contains 36 items,

measuring 8 aspects of health domains: physical functioning, social functioning, physical role

limitations, emotional role limitations, mental health, vitality, bodily pain, and general health

perception.

1. **Multidimensional Fatigue Inventory (MFI; Smets, Garssen, Bonke, & De Haes, 1995)**

MFI is a 20-item self-report instrument designed to measure fatigue. Ratings on a 5-point Likert scale are obtained on the dimensions of general fatigue, physical fatigue, mental fatigue, reduced motivation and reduced activity.

1. **Sheehan Disability Scale (SDS; Sheehan, 1983)**

SDS is a brief, 5-item self-report tool that assesses functional impairment in work/school, social life, and family life
