# Supplementary material for: Effects of a group-based lifestyle medicine for depression: A pilot randomized controlled trial
Source: PLoS One. 2021 Oct 8;16(10):e0258059. doi: 10.1371/journal.pone.0258059 (PMC8500430; doi:10.1371/journal.pone.0258059)
Supplement: S1 File — (PDF) [file pone.0258059.s007.pdf]

THE CHINESE UNIVERSITY OF HONG KONG

Survey and Behavioural Research Ethics

Application Form

NOTE: This Form is to be completed by researchers whose research studies involve survey or observation on human subjects. Please read the ***Guidelines for Survey and Behavioural Research Ethics*** carefully before completing this Form. Once the Form is completed, please submit together with all relevant documents to the *Survey and Behavioural Research Ethics Faculty Sub-committee* (c/o Faculty Office concerned). With all the necessary information and documents received, the processing time of each application is approximately 6 to 8 weeks from the time of application.

**[For SBREC Use Only]**		
Reference No.	:	
Date of Received	:	
Type of Review	:	☐ Expedited
		☐ Full

**Part A: Basic Information**			
**Project Title**<br><br>Lifestyle Medicine for Depression: A Pilot Randomized Controlled Trial			
**Name of Grant Applied /Being Applied**<br>*(if applicable)*<br>N/A		**Grant Ref. No. / Account No.**<br>*(if applicable)*<br>N/A	
**Name of Principal Investigator**<br>*(Please underline the surname)(in full)*<br>Prof. Fiona Y. Y. Ho		**Department / Unit**<br>*(in full)*<br>Psychology	
**Tel. No.**<br><br>3943 3469		**E-mail Address**<br><br>fionahoyy@cuhk.edu.hk	
For students	**Name of Supervisor / Course Instructor**	**Programme and Year of Study**	**Student ID**

Certification of Ethics Training Test(s)

For details, please visit the Research Ethics Training Website (https://www.research-ethics.cuhk.edu.hk/web/)

	E-Cert ID	E-Cert End Date
1. **Survey and Behavioural Research Ethics**	S82200823	21 Aug 2022
2. **Publication Ethics**	P61401682	28 Dec 2022

Part B: Summary of Research Proposal *(maximum 150 words)*
Summarize, in layperson's language, the objective(s) and significance of the research.

This study will examine the feasibility and efficacy of lifestyle medicine for the management of depression in Chinese adult population. The main components of lifestyle intervention typically include physical activity, diet, relaxation/mindfulness, and sleep. While lifestyle medicine has been recognized for centuries as a mean to improve physical health, the field of lifestyle medicine in the context of mental health is still in its infancy (Sarris et al., 2014). In the existing literature, there is

increasing evidence demonstrating the efficacy of individual components of lifestyle medicine (e.g., physical activities and sleep) on depression (Garcia-Toro et al., 2012). However, there is very limited research on the effectiveness of an integration of multiple lifestyle adjustments on depression.

References

García-Toro, M., Ibarra, O., Gili, M., Serrano, M. J., Oliván, B., Vicens, E., & Roca, M. (2012). Four hygienic-dietary recommendations as add-on treatment in depression: a randomized-controlled trial. *Journal of Affective Disorders*, *140*(2), 200-203.

Sarris, J., O'Neil, A., Coulson, C. E., Schweitzer, I., & Berk, M. (2014). Lifestyle medicine for depression. *BMC Psychiatry*, *14*(1), 107.

Part C: Particulars of Research

Human participants

Age range *(Check all that apply)* ☐ 0-17 ☒ 18-59 ☒ 60+

Specification of the inclusion and exclusion criteria of the study *(if any)*

Inclusion criteria:
(1) Hong Kong residents aged ≥ 18 years; (2) Cantonese language fluency; (3) Patient Health Questionnaire (PHQ-9) score ≥ 10; and (4) a willingness to provide informed consent and comply with the trial protocol.

Exclusion criteria:
Participants were excluded if they (1) are pregnant; (2) have suicidal ideation based on Beck Depression inventory (BDI-II) Item 9 score ≥ 2 (referral information to professional services will be provided to those who endorsed items on suicidal ideation); (3) using medication or psychotherapy for depression; (4) having unsafe conditions and are not recommended for exercising or a change in diet by physicians; and (5) major psychiatric, medical or neurocognitive disorders that make participation infeasible or interfere with the adherence to the lifestyle modification.

1. Does the study use **only** publicly available data? ☐ Yes ☒ No

2. Does the study involve **only** survey or observation of public officials? ☐ Yes ☒ No

If you have checked "Yes" to any of the above questions, you can skip Question 3 and go straight to Question 4 to apply for an expedited review.

3. Checklist to determine whether a full review is needed:
   (a) Does the study involve participants who are unable to give informed consent (e.g. children, individuals with intellectual disabilities or cognitive impairments)? ☐ Yes ☒ No

   (If Yes, please elaborate how consent can be obtained in Question 4.)

   (b) Will deception of participants be necessary prior to or during the study? *(If Yes, please attach a debriefing form.)* ☐ Yes ☒ No

   (c) Details on participant remuneration:
   (i) Will participants receive inducements or rewards (other than reasonable expenses and compensation for time) before, during, or after participation? ☐ Yes ☒ No

   If Yes, please specify the form of remuneration e.g. cash, course credit, gift certificate. For cash and gift, state the amount; for course credit, state its percentage of the final grade:

(ii) Will the payment be prorated for partial participation? ☐ Yes ☒ No

(d) Does the study involve sensitive aspects of the participant's own behaviour such as illegal conduct, illicit drug use, suicidality, and sexual conduct? *(If Yes, please provide details in Question 4.)* ☐ Yes ☒ No

(e) If the observations on the participants are disclosed, will it reasonably place the participant at risk of criminal or civil liability or be damaging to the participant's financial standing, employability, or reputation? ☐ Yes ☒ No

If Yes, please provide details below:

(f) Does the study/experiment induce undue psychological stress? ☐ Yes ☒ No

If Yes, please provide details below:

(g) Is pain or more than mild discomfort likely to result from participating the study? ☐ Yes ☒ No

If Yes, please provide details below:

(h) Does the study involve prolonged and repetitive testing? ☒ Yes ☐ No

If Yes, please provide details below:

The participants will complete a set of online questionnaires before the treatment commences, immediately after treatment, and 12 weeks after the treatment sessions are completed.

(i) Will the participants be identified? ☒ Yes ☐ No

If Yes, please provide details below:

Participants will be identified by other participants because participants in the treatment group will receive a group lifestyle medicine treatment.

If you have checked "Yes" to any of the above items, you must go through a full review. Please attach a detailed research proposal.

4. Summary of Research Procedures *(maximum 300 words)*

Describe what the participants will do, where the research activities will take place, the total number of sessions the participants need to participate and their total time commitment. Besides, please attach the instruments (e.g. questionnaire, focus group discussion guide, interview guide, etc.) that will be used in data collection. If the instruments are not yet available, please provide a detailed description of them.

This study will be a pilot randomized controlled trial on the effectiveness of lifestyle medicine for depression. Prior to all study procedures, an online informed consent (with phone support) will be obtained from potential participants. Around 30 eligible participants will be randomly assigned to either the lifestyle medicine group (treatment group) or the Care-As-Usual group (CAU group) in a ratio of 1:1. The randomization will be performed by an independent assessor using a computer-generated list of numbers. No deception is necessary. Participants in the treatment group will receive 2-hour group lifestyle modification at the Chinese University of Hong Kong once per week for 6 consecutive weeks. The group treatment will be primarily delivered by a clinical psychology trainee under the supervision of a clinical psychologist and other healthcare professionals such as a dietitian, a traditional Chinese medicine practitioner, a psychiatrist, and a nurse. The CAU group will not receive the lifestyle medicine treatment, but can continue receiving the routine care as usual. The treatment group will complete a set of online questionnaires before the treatment commences, immediately after treatment, and 12 weeks after the treatment sessions are completed. The CAU group will complete the same set of online questionnaires at the same periods.

5. Will consent form be used? ☒ Yes ☐ No

If No, please provide reason(s) below:

(a) Type of consent *(Check all that apply) (Please attach a copy of consent form.)*

☒ Formal Consent

☒ Informed consent
(for participants able to give legal valid informed consent and not belonging to any vulnerable groups)

☐ Parent/guardian consent
(for participants under 18 years old or those belonging to a vulnerable group)

☐ Assent form
(for participants not competent to give legal valid informed consent)

☐ Passive informed consent
(for surveys to be conducted through the schools or other authorities)

☐ Informal Consent

Please provide justifications:

6. Confidentiality of data

(a) Collection of auditory and visual data *(Check all that apply)*

☐ Audiotapes ☐ Still photos
☐ Videotapes ☒ Not applicable

(b) Does the study involve multi-phase data collection? ☒ Yes ☐ No

If Yes, please explain the tracking and coding system(s):
The participants will complete a set of online questionnaires before the treatment commences, immediately after treatment, and 12 weeks after the treatment sessions are completed.

(c) Does the study involve collecting data through an Internet ☒ Yes ☐ No

survey platform?

If Yes, please address the confidentiality of data collected via e-mail, Web interfaces, and other networked information:

Outcome data will be collected using a smartphone application. Participant's personal information will be sent to a secured server. The passwords created by the participants and the collected data will be encrypted using MD5, a message-digest algorithm that is widely used as a cryptographic hash function. Participants are required to log into the system before they can use any functions or features in the app.

(d) Will any individually identifiable information, including images of participants, be published, shared, or disseminated? ☐ Yes ☒ No

If Yes, please elaborate how the explicit consent or assent for such publication/share/dissemination can be obtained from participants:

7. Data Security

Describe how and where data will be kept to protect participant's confidentiality.

Identifying information will be removed from the data file and stored separately, with the link between identifying information and data made through codes only. Any entered data collected from participants will only be accessed and stored via a password-protected computer. All electronic copies of the data will be stored on a password-protected computer or password-protected USB drive which will be kept by the student investigator at all times or stored in a securely locked location.

DECLARATION:

(i) The information provided above is to the best of my knowledge accurate. I shall take reasonable care to ensure that the project is conducted in accordance with the *Guidelines for Survey and Behavioural Research Ethics*. I will obtain approval from other responsible units within CUHK (e.g. The Joint Chinese University of Hong Kong (CUHK) Hospital Authority New Territories East Cluster (NTEC) Clinical Research Ethics Committee (CREC), Animal Experimentation Ethics Committee (AEEC), University Safety Office/University Laboratory Safety Office) where appropriate.

(ii) I ascertain that:

- permission from the copyright holder(s) has been/will be obtained for the use of either the original or derivative version of any copyright protected tests or assessment instruments, and
- payment for the use of the test(s) (if any) has been/will be made.

**Date**	**Signature of Principal Investigator**
23-09-2018	[signature]

For students

ENDORSEMENT BY SUPERVISOR / COURSE INSTRUCTOR

**Date**	**Signature of Supervisor / Course instructor**

Please submit the application to:

- Faculty Sub-committee (c/o Faculty Office concerned)
  (for Faculties of Arts, Business Administration, Social Science, Medicine, Law, and Education)
- Survey and Behavioural Research Ethics Committee (c/o Faculty Office of Social Science)
  (for Faculties of Engineering, and Science)

Submission Checklist – Supporting Documents *(if applicable)*

This checklist is provided to assist the Principal Investigator to submit a SBRE application. Please ensure the checklist has been completed prior to submitting the application.

☐ Research proposal (for application requiring a full review)

☒ Instruments for data collection

☐ Debriefing form

☒ Informed consent document(s)

☐ Other documents, please specify:

____

____

For use of the *Survey and Behavioural Research Ethics Committee/Faculty Sub-Committees:*
(Please tick as appropriate)

1. First screening by Faculty Sub-Committee:

Expedited review: ☐ Yes ☐ No, a full review is needed

If the application meets the requirements for expedited review:
Approval of application: ☐ Yes ☐ No

Further Actions required: ______________________

Signature : ______________________

Name : ______________________

Date : ______________________

2. For application which needs full review:

Recommendation by Full Reviewer of Faculty Sub-committee	Recommendation by Chairperson of the Survey and Behavioural Research Ethics Committee
Full review: ☐ Yes ☐ No	Full review: ☐ Yes ☐ No
Approval of application: ☐ Yes ☐ No	Approval of application: ☐ Yes ☐ No
Remarks : ______________	Remarks : ______________
Signature : ______________	Signature : ______________
Name : ______________	Name : ______________
Date : ______________	Date : ______________
